# Modified Pechini-derived Na_3_V_2_(PO_4_)_3_/C with superior low-temperature performance for sodium-ion batteries

**DOI:** 10.1039/d5ra08915h

**Published:** 2026-01-21

**Authors:** Ilyas Mukushev, Nurbolat Issatayev, Aliya Mukanova, Zhanar Jakupova, Zhumabay Bakenov, Kim Sung-Soo, Arailym Nurpeissova

**Affiliations:** a National Laboratory Astana, Laboratory of Energy Storage Systems Kabanbay Batyr Ave. 53 Astana 010000 Kazakhstan arailym.nurpeissova@nu.edu.kz; b Institute of New Materials and Energy Technologies Kabanbay Batyr Ave. 53 Astana 010000 Kazakhstan; c Department of Chemistry, L.N. Gumilyov Eurasian National University Satpayev St. 2 Astana 010000 Kazakhstan; d Institute of Batteries Kabanbay Batyr Ave. 53 Astana 010000 Kazakhstan; e Department of Chemical and Materials Engineering, School of Engineering and Digital Sciences, Nazarbayev University Kabanbay Batyr Ave. 53 Astana 010000 Kazakhstan; f Graduate School of Energy Science and Technology, Chungnam National University 99 Daehak-ro, Yuseong-gu Daejeon 34134 South Korea kimss@cnu.ac.kr

## Abstract

Sodium-ion batteries (SIB) have attracted significant attention as promising alternatives to lithium-ion batteries, due to the abundance, low cost, and environmental compatibility of sodium resources. Among various SIB cathode materials, NASICON-type Na_3_V_2_(PO_4_)_3_ (NVP) stands out for its stable 3D framework and fast Na^+^ diffusion kinetics. However, its inherently low electronic conductivity has posed challenges for practical implementation. Promising in this context, the NASICON-structure NVP nanomaterial was successfully synthesized using a modified Pechini method. Owing to the reduced particle size and carbon coating, the cathode exhibits exceptional performance, demonstrating a capacity of 74.13 mAh g^−1^ at 0.2 C even at −20 °C, which is 76.26% of its room-temperature capacity. Furthermore, the electrode retained 74.99% of its room-temperature capacity at −25 °C, demonstrating outstanding low-temperature resilience. These results confirm that the modified Pechini-derived NVP/C composite is a highly promising cathode material for high-performance sodium-ion batteries operating under subzero conditions.

## Abstract 2

NASICON-type Na_3_V_2_(PO_4_)_3_/C synthesized by a modified Pechini method shows excellent low-temperature performance for sodium-ion batteries, delivering 74.13 mAh g^−1^ at −20 °C (76.26% retention) and maintaining 74.99% capacity at −25 °C, demonstrating strong subzero resilience.

## Graphical abstract

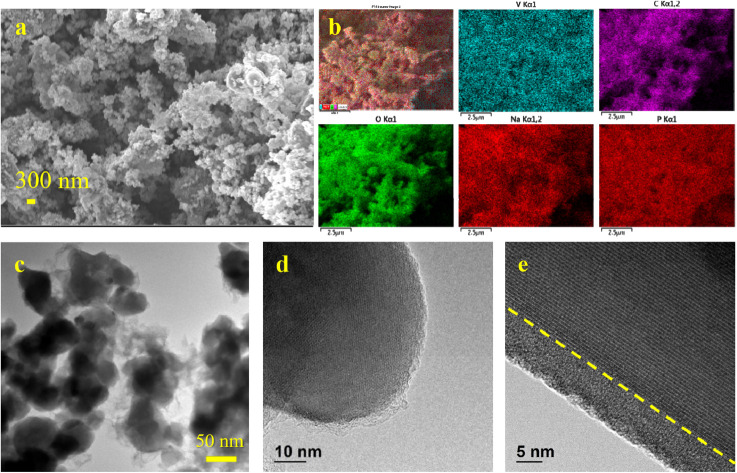

## 1 Introduction

Sodium-ion batteries (SIBs) have garnered growing interest as cost-effective and sustainable alternatives to lithium-ion systems due to the abundance and low cost of sodium, as well as compelling progress in cathode/anode materials and cell engineering.^1–3^ However, low temperatures (LTs) severely degrade the performance of SIBs and constrain their use in high-altitude and cold-climate applications, posing challenges for aerospace, hybrid electric vehicles (HEVs), and electric vehicles (EVs).^4,5^ The principal cause of LT performance loss is the temperature-dependent change in liquid–electrolyte properties (increased viscosity, altered solvation, and reduced ionic conductivity) and the formation/behavior of the interphase, which together impede Na^+^ transport between cathode and anode and slow interfacial charge transfer, thereby reducing reaction rates.^6,7^ Yet improving the electrolyte alone is often insufficient, because electrode intrinsic kinetics, notably sluggish Na^+^ diffusion in bulk host structures and poor electronic conductivity, also limit LT behavior.^8^ To compensate for reduced Na^+^ conductivity and slow interfacial kinetics at low temperatures, cathodes should be engineered with small particle sizes, conductive carbon coatings, and architectures that enhance both ionic and electronic transport.^9^

Among reported sodium cathodes, polyanionic NVP and layered Na_*x*_TMO_2_ (P2/O3-type) compounds exhibit excellent room-temperature performance. Nevertheless, their LT capabilities are often hampered by sluggish Na^+^ migration within 1D/2D transport channels and interfacial kinetic barriers.^10^ Careful compositional and doping strategies for layered P2-type cathodes (*e.g.*, Nb-doping) have been shown to strongly affect LT kinetics: a Nb-doped P2 cathode was reported to retain high capacity even at −40 °C, whereas less optimized layered materials show much larger losses at subzero temperatures – highlighting that both microstructure and chemistry must be tuned for operability in cold conditions.^11^

In recent years, extensive efforts have been devoted to improving the electrochemical performance of sodium-ion battery cathodes through compositional engineering, including cation/anion doping, defect and vacancy regulation, and interfacial modification. For example Haofei *et al.* achieve 76% capacity retention after 200 cycles at 990 mA g^−1^ by employing oxygen vacancy engineering induced by binary metal–ion (Co^2+^/Ni^2+^) pre-intercalation.^12^ For instance, oxygen-vacancy engineering combined with Co^2+^ pre-intercalation and Ti_3_C_2_T_*x*_ surface coating has enabled layered HNaV_6_O_1_·4H_2_O cathodes—previously ineffective for sodium-ion batteries – to achieve discharge capacities above 80 mAh g^−1^ with ∼75–79% capacity retention after prolonged cycling at high current densities.^13^ Similarly, local electronic structure regulation through alkali-metal substitution has been shown to effectively suppress structural distortion in layered vanadium oxides, enabling outstanding cycling stability at high current densities.^14,15^ Recent reviews on aqueous zinc–inorganic batteries have highlighted that proton (H^+^) storage, enabled by fast hopping and Grotthuss transport mechanisms and regulated through interlayer, doping, defect, and composite engineering, can exhibit superior reaction kinetics compared with multivalent-ion storage, providing valuable insights into ion-transport modulation in polyanionic and layered cathode materials.^16^

In contrast to many layered oxide cathodes with one- or two-dimensional Na^+^ diffusion pathways, the polyanionic compound NVP features a three-dimensional (3D) NASICON-type framework that enables rapid sodium-ion transport and excellent structural stability.^17,18^

For example, NVP with nanoscale carbon coating and reduced particle dimensions shows markedly improved low-temperature rate and capacity: an NVP@C variant delivered 81.9 mAh g ^−1^ at 100 mA g ^−1^ and 60.2 mAh g ^−1^ at 1000 mA g ^−1^ at −15 °C, demonstrating the value of particle-size and conductivity optimizations.^19^

These characteristics make NVP highly attractive for LT SIBs, where sluggish ion diffusion and interfacial charge-transfer kinetics typically limit performance.^5,8^ Moreover, its robust open framework and minimal volume change during Na^+^ insertion/extraction contribute to long cycle life and high-rate capability.

In recent studies, additional improvements in NVP performance have been achieved by tuning synthesis parameters such as calcination temperature, carbon precursor ratio, and annealing atmosphere.^20^ To further enhance electronic conductivity and Na^+^ transport, composite strategies and particle-size optimization have been widely employed. For example, Li *et al.* prepared NVP/C nanoplates *via* a hydrothermal method followed by post-calcination, delivering a reversible capacity of 117 mAh g^−1^ at 0.2 C, maintaining a high-rate performance of 76.5 mAh g^−1^ at 100 C, and exhibiting exceptionally long cycling stability.^21^ In addition, reducing particle size through modified Pechini and polymer-assisted sol–gel methods improves both Na^+^ diffusion and electrical conductivity by forming uniform carbon coatings that prevent particle agglomeration.^22,23^

These structural and morphological features are crucial for maintaining performance at LTs. Indeed, NASICON-type analogues such as rhombohedral Li_3_V_2_(PO_4_)_3_ (R-LVP) have demonstrated outstanding LT properties. Qin *et al.*^23^ reported that R-LVP retained 90 mAh g^−1^ at −30 °C, about 80% of its room-temperature capacity, emphasizing the inherent advantages of this framework for LT operation. Consequently, NVP synthesized through the Pechini route, characterized by its 3D Na^+^ conduction channels, excellent electronic conductivity, and reduced particle size, represents an up-and-coming cathode candidate for high-rate and LT SIBs.

In this study, rhombohedral NVP/C was synthesized using a modified Pechini method and systematically evaluated, for the first time, as an LT cathode material for SIBs. The electrochemical performance, structural features, and surface chemical characteristics of the NVP/C electrode were comprehensively investigated. The NVP/C electrode exhibited outstanding rate capability, delivering discharge capacities of 83.67 mAh g^−1^ at 5 C, 75.39 mAh g^−1^ at 10 C, and 50.27 mAh g^−1^ at 20 C. Even after 1000 cycles at 1 C at RT, 95% of the initial capacity was retained, indicating superior structural stability and high reversibility. Furthermore, at −20 °C, a discharge capacity of 74.13 mAh g^−1^ was achieved at 0.2 C, corresponding to 76.26% retention relative to RT performance and even slightly increased after 100 cycles. These results confirm that the modified Pechini-derived NVP/C composite possesses excellent ionic/electronic conductivity and outstanding low-temperature stability, making it a highly promising cathode for next-generation sodium-ion batteries.

## 2 Experimental part

### 2.1 Synthesis of Na_3_V_2_(PO_4_)_3_/C

The NVP/C cathode material was synthesized *via* a modified Pechini method,^23^ following the same procedure previously used in our earlier study with a Li anode.^24^ The technique relies on the polycondensation reaction between ethylene glycol and citric acid, forming a polymeric network that inhibits particle growth during calcination. Oxalic acid and citric acid served as both reducing and chelating agents as well as the carbon source.

Analytical-grade V_2_O_5_ (Sigma-Aldrich, 98%), oxalic acid (99%), citric acid (99%), and NaH_2_PO_4_ (99%) were used in a molar ratio of 1 : 6:6 : 3. Each reagent was first dissolved separately in a water–ethylene glycol mixture (3 : 7 volume ratio) and then combined under continuous stirring. Upon addition of oxalic and citric acids to V_2_O_5_, the solution's color changed from yellow to blue, indicating the reduction of vanadium from V^5+^ to V^3+^.

The resulting homogeneous mixture was maintained at 80 °C for 7 h, followed by preheating at 300 °C for 2 h in a muffle furnace. The obtained precursor was ground into a fine powder and then annealed in an argon atmosphere at 600 °C for 10 h and subsequently at 800 °C for 2 h using a tube furnace to yield the final NVP/C composite.

### 2.2 Materials characterization

The phase composition of all samples was examined using X-ray diffraction (XRD) on a Rigaku Miniflex 600 diffractometer. The obtained diffraction patterns were refined *via* the Rietveld method using SmartLab Studio II (Rigaku) software. The morphology and microstructure were characterized by scanning electron microscopy (SEM, Carl Zeiss Crossbeam 540) and spherical aberration–corrected scanning transmission electron microscopy (Cs-STEM, JEOL JEM-ARM200F). The elemental distribution was analyzed using SEM (JEOL JSM-IT200(LA)) equipped with an energy-dispersive X-ray spectroscopy (EDS) detector.

The Raman spectra were recorded with a Horiba LabRAM spectrometer employing a 532 nm excitation laser. The carbon content in the composites was quantified using a CHNS elemental analyzer (vario MICRO cube, Elementar). The surface chemical states were investigated by X-ray photoelectron spectroscopy (XPS) using a Thermo Scientific NEXSA instrument operating under ultra-high vacuum conditions with a monochromatic Al Kα X-ray source (1486.6 eV) and a 180° double-focusing hemispherical analyzer equipped with a 128-channel detector. The XPS spectra were processed and fitted using Thermo Avantage software.

### 2.3 Electrochemical tests

The working electrodes were fabricated by casting a homogeneous slurry composed of 80 wt% NVP/C, 10 wt% polyvinylidene fluoride (PVDF), and 10 wt% Super P conductive carbon dispersed in *N*-methyl-2-pyrrolidone (NMP) onto aluminum foil as the current collector. The coated electrodes were then dried under vacuum at 80 °C for 12 h. The active material loading was determined using an Ultramicrobalance (XP Micro, Mettler Toledo) and adjusted to approximately 2 mg cm^−2^.

The coin cells (CR2032) were assembled in an argon-filled glove box (Mbraun, Labmaster Pro DP) for NVP//Na with Na metal as anode and counter electrode, Whatman GF/D glass microfiber filters as separator and 0.5M NaPF_6_ in a mixture of propylene carbonate (PC) and fluoroethylene carbonate (FEC) (98 : 2 in vol.ratio) as electrolyte.

Galvanostatic charge–discharge tests were performed within a voltage window of 2.7–3.8 V *vs.* Na^+^/Na at various C-rates using a Neware battery testing system, both at RT and −20 °C. Cyclic voltammetry (CV) measurements were conducted using a Biologic VMP3 electrochemical workstation in the potential range of 2.7–3.8 V at a scan rate of 0.1 mV s^−1^.

## 3 Results and discussion

**Fig. 1:**
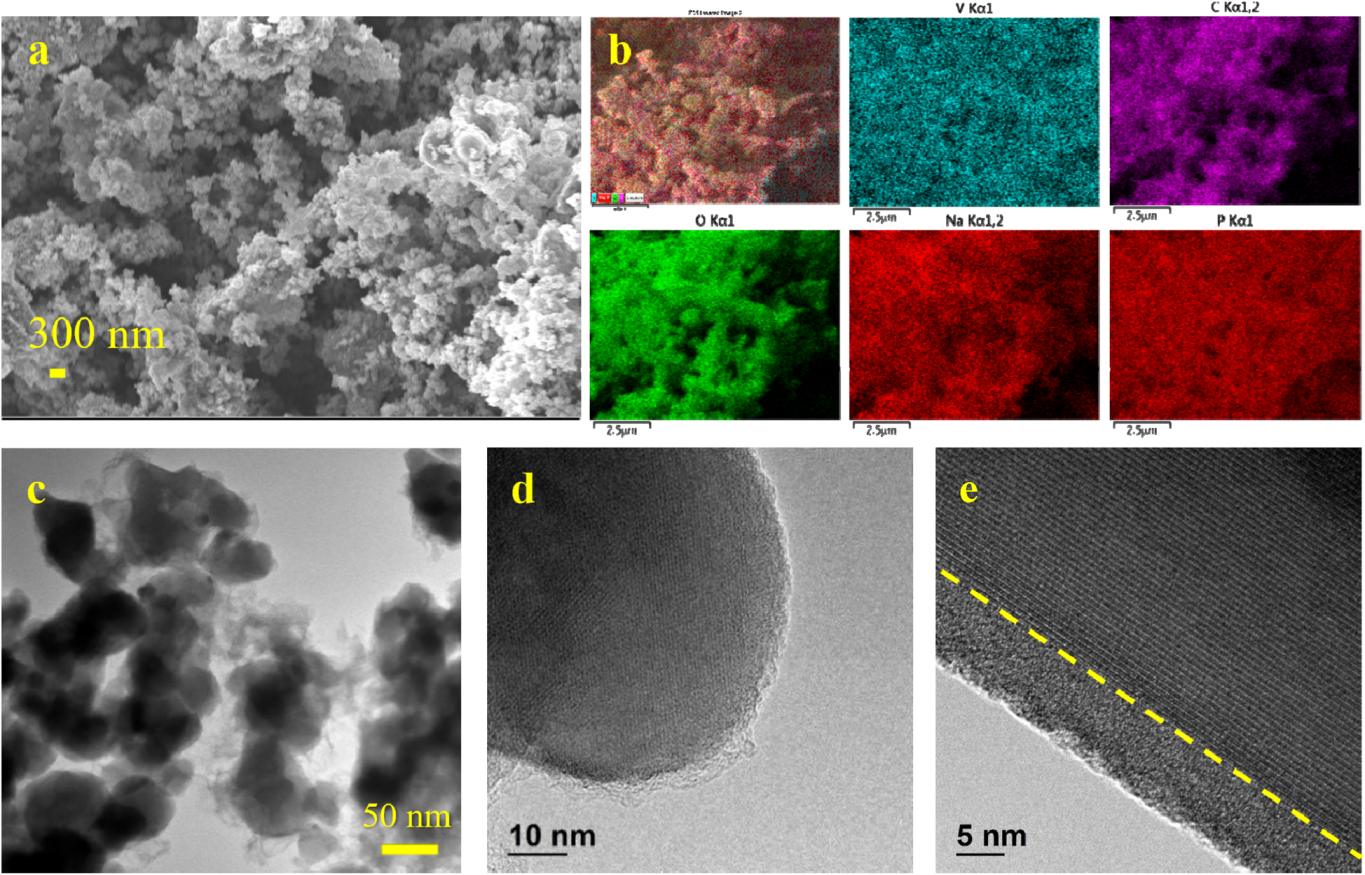
(a) SEM image, (b) EDS map, (c) TEM image, and (d and e) HRTEM images of NVP/C powder.

The morphology, elemental distribution, and microstructure of the synthesized NVP/C powder were examined using SEM, EDS, and TEM analyses. The SEM image (Fig. 1a) shows uniformly distributed, nearly spherical nanoparticles with sizes ranging from 50 to 150 nm. Such morphology and fine particle size result from the polycondensation reaction between ethylene glycol and citric acid in the modified Pechini process, which effectively restricts particle growth and is expected to enhance low-temperature electrochemical performance.^25^

The EDS elemental mapping (Fig. 1b) confirms the homogeneous distribution of Na, V, P, O, and C throughout the composite, indicating uniform composition of the active material.

The TEM observations (Fig. 1c) further verify that the NVP particles are uniformly encapsulated by a carbon matrix, forming well-defined core–shell structures. High-resolution TEM images (Fig. 1d and f) reveal a continuous carbon coating layer approximately 12 nm thick, which originates from the relatively high molar ratios of citric and oxalic acids used in the Pechini route. The interface between the NVP cathode particles and the carbon coating is highlighted by dashed lines (Fig. 1f) to clearly distinguish the carbon layer from the active material. This conductive carbon layer ensures efficient electron transport and structural stability during cycling. Additionally, the clear lattice fringes observed in the HRTEM images demonstrate the high crystallinity of the synthesized NVP/C, with an interplanar spacing of 0.624 nm corresponding to the (012) plane of rhombohedral NASICON-type NVP.^26^

**Fig. 2:**
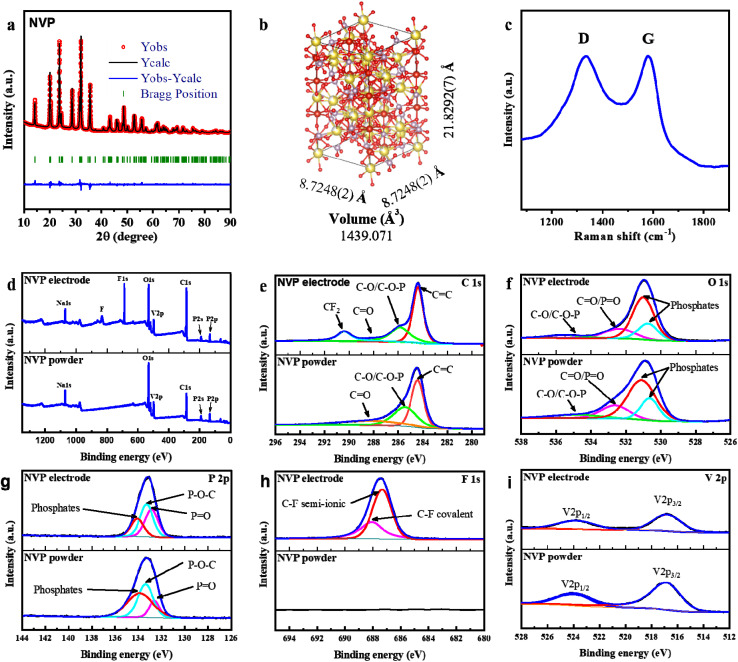
(a) Rietveld-refined XRD patterns of the NVP/C, where observed data are indicated by red circles, the calculated pattern by a black line, and the difference pattern by blue. The residual discrepancy is shown in blue. (b) Illustration of the crystal structure for the NVP created by VESTA software. (c) Raman spectra of the NVP/C. (d) Survey XPS spectra and deconvoluted XPS spectra of (e) C 1s, (f) O 1s, (g) P 2p, (h) F 1s, and (i) V 2p of NVP/C powder and electrode.

The XRD analysis was conducted to evaluate the phase purity and crystallinity of the pristine NVP/C powder (Fig. 2a). The diffraction peaks of the pristine NVP/C powder are in good agreement with the reference PDF card no. 00-062-0345 of Na_3_V_2_(PO_4_)_3_, confirming the formation of a pure phase without any detectable impurities.^27^ The refined lattice parameters obtained from Rietveld analysis (Fig. 2a and b) also confirm the successful synthesis of NVP/C.

According to CHNS analysis, the carbon content in the NVP/C composite was determined to be approximately 13 wt%. This relatively high value is associated with the elevated molar ratio of citric and oxalic acids used during synthesis, which serve as carbon precursors. The Raman spectrum of NVP/C (Fig. 2c) displays two prominent peaks at 1335 and 1586.5 cm^−1^, corresponding to the D band (disordered carbon) and G band (graphitic sp^2^ carbon), respectively.^28^ The intensity ratio of these two peaks (*I*_D_/*I*_G_ = 1) suggests a well-balanced coexistence of amorphous and graphitic carbon structures, which can facilitate both electronic conductivity and structural stability of the composite.^29,30^

To investigate the surface chemical composition NVP/C was analyzed using X-ray photoelectron spectroscopy (XPS). As expected, the XPS survey spectrum (Fig. 2d) shows that the NVP/C contains Na, O, V, P and C. The high-resolution C 1s spectrum (Fig. 2e) exhibits three distinct peaks at 284.42, 285.44, and 288.13 eV, corresponding to C=C, C–O/C–O–P, and C=O bonds, respectively.^31^

**Fig. 3:**
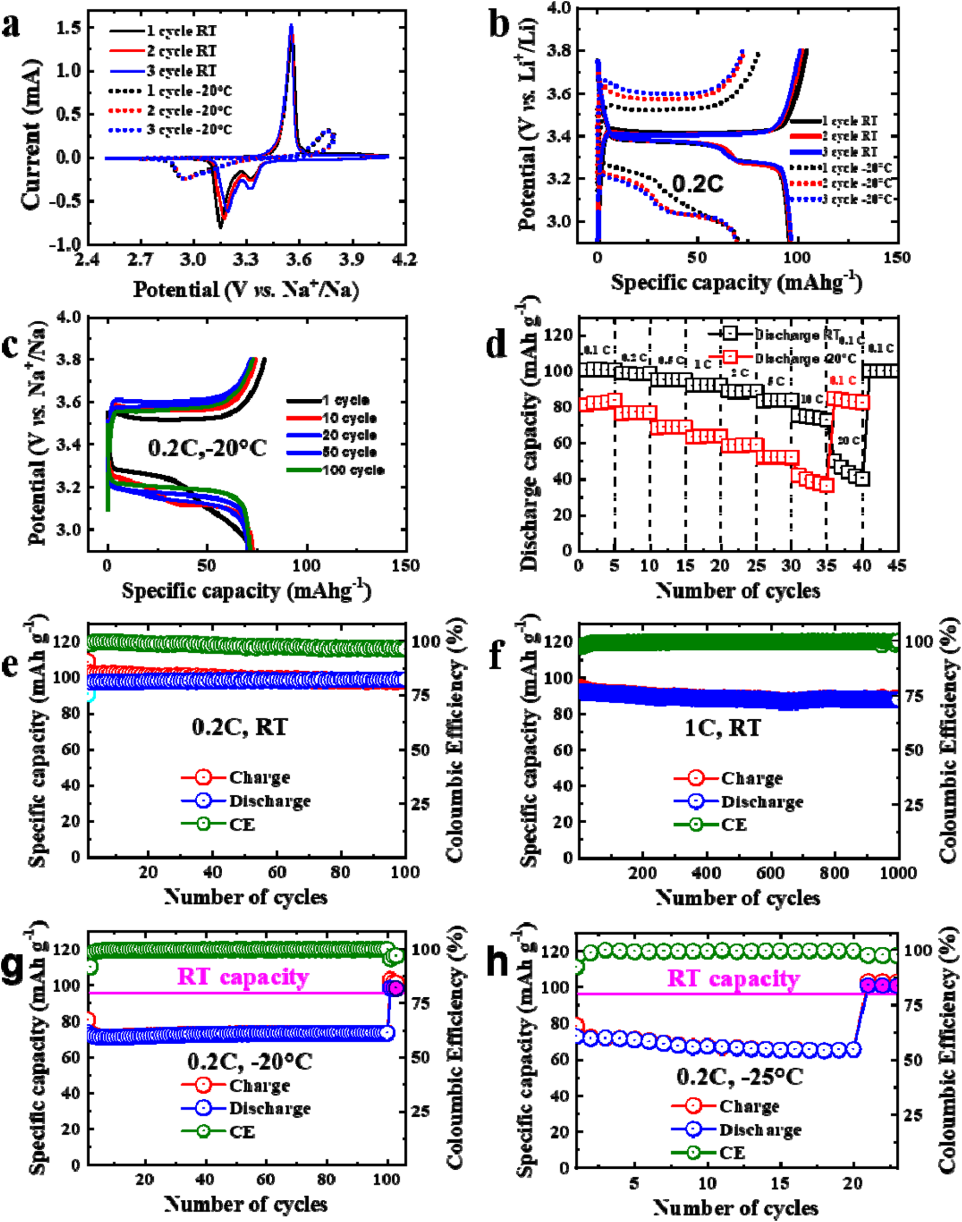
(a) CV scans of the NVP/C at a scan rate of 0.1 mV s^−1^ at RT and −20 °C; (b) Galvanostatic charge/discharge curves for three cycles of the NVP/C in the potential range of 2.7–3.8 V *vs.* Na^+^/Na at 0.2 C at RT and −20 °C, and (c) for cycles 1–100 at −20 °C; (d) rate performance of the NVP/C at RT and −20 °C. Cycling performance of the NVP/C at RT at (e) 0.2 and at (f) 1 C. Cycling performance of the NVP/C at (g) −20 °C and −25 °C at (h) 0.2 C.

The deconvoluted O 1s spectrum (Fig. 2f) further confirms the presence of oxygenated surface species. Peaks located at 532.62 and 534.9 eV are assigned to C–O/C–O–P and C=O/P=O bonds, respectively, while those at 530.63 and 531.1 eV indicate the presence of phosphate groups in the NVP/C framework.^32^ The P 2p spectrum (Fig. 2g) shows a dominant peak at 133.37 eV corresponding to the P–O–C bond, with additional components at 132.62 eV and 133.86 eV attributed to P=O and PO_4_^3−^ moieties, respectively. The high-resolution V 2p spectrum (Fig. 3i) displays two characteristic peaks at 517.07 eV and 524.2 eV, corresponding to V 2p_3/2_ and V 2p_1/2_ transitions, confirming that vanadium mainly exists in the V^3+^ oxidation state within the NASICON structure.^33^

The well-preserved oxidation state of vanadium further demonstrates that the synthesis and electrode preparation processes do not induce undesired surface reduction or phase transformation, confirming the chemical robustness of the NVP/C cathode. Overall, these results verify the chemical integrity and surface stability of the NVP/C cathode before electrochemical cycling.

Electrochemical measurements were conducted to evaluate the performance of the NVP/C cathode under different temperature conditions. As shown in Fig. 3a, the cyclic voltammetry (CV) curves of the NVP/C//Na at RT display an oxidation peak at approximately 3.55 V, along with two overlapping reduction peaks at around 3.15 V and 3.32 V. One main oxidation peak at ∼3.55 V, corresponding to the oxidation of V^3+^ → V^4+^ during Na^+^ extraction from the NASICON framework (de-sodiation process). Two overlapping reduction peaks at ∼3.32 V and ∼3.15 V, which indicate two-step Na^+^ insertion processes accompanied by the reduction of V^4+^ → V^3+^. The stable appearance of these redox peaks suggests highly reversible Na^+^ intercalation within the NASICON framework.

At −20 °C, the NVP/C//Na cell exhibits one oxidation peak at ∼3.76 V and a reduction peak at ∼2.94 V, indicating that the redox potentials shift toward higher polarization at low temperatures and that the reduction peaks overlap completely. This increased overpotential can be attributed to the reduced ionic conductivity of the electrolyte and the slower electrochemical kinetics at subzero temperatures.^4,34^

Fig. 3b presents the initial charge–discharge curves of the NVP/C//Na cell in the potential range of 2.7–3.8 V at 0.2 C at RT and −20 °C. The discharge plateaus at RT observed around 3.4 V correspond to the reversible V^3+^/V^4+^ redox couple associated with Na^+^ insertion/extraction within the NASICON-type NVP framework. The cell delivers an initial charge capacity of approximately 104.4 mAh g^−1^ and a reversible discharge capacity of 97.21 mAh g^−1^ at 0.2 C, which is comparable to previously reported NVP-based cathodes operating within a similar voltage window.^35^ Although a higher capacity can be achieved by expanding the voltage range, such an approach is often accompanied by deteriorated cycling stability and reduced capacity retention.^36,37^ The capacity loss during the first cycle is likely associated with electrolyte decomposition and CEI layer formation.^38,39^

Fig. 3c shows the charge–discharge profiles at −20 °C at 0.2 C. The initial charge curve begins at ∼3.52 V and then stays about ∼3.56 V, indicating strong polarization due to limited ionic transport at low temperatures. Polarization decrease during low-temperature cycling can be attributed to the gradual electrochemical activation of the electrodes, stabilization of the CEI layer, and improved interfacial charge transfer kinetics upon repeated cycling.^5,40^

The rate performance of the NVP/C at RT is displayed in Fig. 3d. The cathode delivers discharge capacities of 100.56, 98.79, 95.25, 92.06, 88.93, 83.41, 75.39, and 50.27 mAh g^−1^ at rates of 0.1, 0.2, 0.5, 1, 2, 5, 10, and 20 C, respectively. At −20 °C, the NVP/C electrode exhibits discharge capacities of 81.03, 76.42, 68.53, 63.22, 58.51, 52.15, and 42.29 mAh g^−1^ at 0.1, 0.2, 0.5, 1, 2, 5, and 10 C, respectively, corresponding to 83.36, 78.61, 70.5, 60.2, 53.65, and 43.5% of the RT capacity at 0.2 C. When the current rate is returned to 0.1 C, the capacity is fully recovered, confirming excellent structural reversibility and high-rate stability.

Fig. 3e and f show the cycling performance of the NVP/C//Na at 0.2 C during 100 cycles and 1 C during 1000 cycles at RT. At 0.2 C, the discharge capacity even slightly increases from 97.51 mAh g^−1^ to 98.65 mAh g^−1^ after 100 cycles. Good capacity retention after C-rate and discharge capacity increase as well as polarization decrease during low-temperature cycling can be explained by activation of the electrode, stabilization of the CEI layer, and improved interfacial charge transfer kinetics during cycling.^5,40^ At 1 C, the discharge capacity even decreases slightly from 92.2 mAh g^−1^ to 87.59 mAh g^−1^ after 1000 cycles, corresponding to only a 5% capacity loss. As shown in Fig. 3g, when cycled at −20 °C, the discharge capacity decreases from 74.13 mAh g^−1^ (76.26% of RT capacity) in the first cycle to 73.6 mAh g^−1^ (75.71%) after 100 cycles. At −25 °C (Fig. 3h), the capacity decreases from 72.9 mAh g^−1^ (74.99%) to 65.67 mAh g^−1^ (67.55%) after 20 cycles. The greater capacity fading at lower temperatures can be mainly attributed to the changes in the electrolyte's physical properties, such as increased viscosity and partial freezing, which restrict Na^+^ transport and slow down charge-transfer kinetics.^4,5^

After tests at −20 °C and −25 °C, the coin cells were again tested at RT and showed excellent capacity retention. Good battery performance can be partially attributed to the FEC additive in the electrolyte, which improves electrochemical performance by stabilizing the cathode–electrolyte interphase (CEI), thereby reducing interfacial resistance and polarization, particularly at low temperature.^4,5^ Overall, the good electrochemical performance of the NVP/C//Na at both RT and LT can be attributed to the 3D structure and small particle size, which facilitate ion transportation and carbon coating, which increases electronic conductivity.

## 4 Conclusion

NASICON-structured Na_3_V_2_(PO_4_)_3_ (NVP/C) was successfully synthesized *via* a modified Pechini method and systematically evaluated as a cathode material for sodium-ion batteries. The as-prepared NVP/C exhibited uniformly dispersed, rounded nanoparticles with a continuous carbon coating layer that enhanced both electronic conductivity and structural stability. Structural and surface analyses (XRD, Raman, and XPS) confirmed the formation of a pure rhombohedral NASICON phase with well-preserved phosphate and vanadium frameworks.

Electrochemical testing revealed outstanding rate capability, cycling stability, and excellent low-temperature performance. At room temperature, the NVP/C electrode delivered discharge capacities of 83.41, 75.39, and 50.27 mAh g^−1^ at 5 C, 10 C, and 20 C, respectively, while retaining 95% of its initial capacity after 1000 cycles at 1 C. Even at subzero temperatures, the electrode maintained 74.13 mAh g^−1^ at −20 °C and 72.9 mAh g^−1^ at −25 °C (76.26% and 74.99% of RT capacity), demonstrating stable operation and minimal degradation during prolonged cycling. Notably, after returning to room temperature, the cell fully recovered its initial capacity, confirming excellent structural reversibility.

These results clearly show that the modified Pechini-derived NVP/C composite—characterized by its 3D NASICON framework, nanosized particles, and robust carbon coating—possesses outstanding ionic/electronic conductivity and exceptional low-temperature resilience. The ability of this material to sustain high electrochemical performance even at −25 °C underscores its strong potential as a next-generation cathode for SIB operating in extreme cold or high-altitude environments.

## Author contributions

Ilyas Mukushev: writing – original draft preparation; visualization; investigation; experiments; conceptualization; data curation. Nurbolat Issatayev: conceptualization; data curation, analysis, proofreading and editing. Aliya Mukanova: writing – review and editing. Zhanar Jakupova: conceptualization; supervision. Zhumabay Bakenov: analysis, resources, proofreading and editing. Kim Sung-Soo: conceptualization; supervision; analysis. Arailym Nurpeissova: conceptualization; supervision; resources; funding acquisition; analysis; writing – review and editing.

## Conflicts of interest

The authors declare no conflicts of interest.

## Data Availability

All data supporting the findings of this study are available within the article.

## References

[cit1] Domalanta M. R., Castro M. T., Del Rosario J. A., Ocon J. D. (2022). Cost analysis of a sodium-ion battery pack for energy and power applications using combined multi-physics and techno-economic modeling. Chem. Eng. Trans..

[cit2] Annu A., Harisha B. S., Yewale M., Akkinepally B., Shin D. K. (2025). Green batteries: A sustainable approach towards next-generation batteries. Batteries.

[cit3] Karabelli D., Singh S., Kiemel S., Koller J., Konarov A., Stubhan F., Miehe R., Weeber M., Bakenov Z., Birke K.P. (2020). Sodium-based batteries: In search of the best compromise between sustainability and maximization of electric performance. Frontiers in Energy Research.

[cit4] Bai Z., Yao Q., Wang M., Meng W., Dou S., Liu H. K., Wang N. (2024). Low-temperature sodium-ion batteries: challenges and progress. Adv. Energy Mater..

[cit5] Zhao Y., Zhang Z., Zheng Y., Luo Y., Jiang X., Wang Y., Wang Z., Wu Y., Zhang Y., Liu X., Fang B. (2024). Sodium-ion battery at low temperature: Challenges and strategies. Nanomaterials.

[cit6] Li P., Hu N., Wang J., Wang S., Deng W. (2022). Recent progress and perspective: Na ion batteries used at low temperatures. Nanomaterials.

[cit7] Lutsenko D. S., Belova E. V., Zakharkin M. V., Drozhzhin O. A., Antipov E. V. (2023). Low-Temperature Properties of the Sodium-Ion Electrolytes Based on EC-DEC, EC-DMC, and EC-DME Binary Solvents. Chemistry.

[cit8] Zeng X., Peng J., Guo Y., Zhu H., Huang X. (2020). Research progress on Na_3_V_2_ (PO_4_) 3 cathode material of sodium ion battery. Front. Chem..

[cit9] Hu J., Li X., Liang Q., Xu L., Ding C., Liu Y., Gao Y. (2025). Optimization Strategies of Na 3 V_2_(PO_4_)_3_ Cathode Materials for Sodium-Ion Batteries. Nano-Micro Lett..

[cit10] Zeng X., Peng J., Guo Y., Zhu H., Huang X. (2020). Research progress on Na3V2 (PO4) 3 cathode material of sodium ion battery. Front. Chem..

[cit11] Shi Q., Qi R., Feng X., Wang J., Li Y., Yao Z., Wang X., Li Q., Lu X., Zhang J., Zhao Y. (2022). Niobium-doped layered cathode material for high-power and low-temperature sodium-ion batteries. Nat. Commun..

[cit12] Yang H., Li W., Dong Q., Zhang J., Jiang Q., Zuo J., Li M., Wang J., Luo Y., Song X., Wang J. (2025). Oxygen Vacancy Engineering Induced by Binary Metal Ions Pre-Intercalation to Realize Stable Na+ Storage at High Voltage. Small.

[cit13] Yang H., Li W., Luo Y., Jia S., Zhang J., Yuan Y., Li X. (2023). Hydrogen Sodium Vanadium Oxide Hydrate with Oxygen Vacancy: A New Layered Cathode Material for Sodium-Ion Battery. ACS Sustain. Chem. Eng..

[cit14] Song X., Wang J., Li W., Xiao W., Zhang G., Liu S., Xu Y., Lu J., Li X. (2025). Local electronic regulation
of Na5V12O32 cathode suppressing structural distortion toward enhanced sodium storage. Nat. Commun..

[cit15] Li Y., Li W., Yang H., Zhang T., Jiang Q., Wang J., Wen H., Li M., Jia W., Hua X., Li X. (2025). Understanding lithium-rich manganese-based cathode materials from the perspectives of lattice challenges and doping engineering. Sci. China: Chem..

[cit16] Li W., Song Q., Dong Q., Zhang J., Wang J., Wu Y., Yu Y., Li X. (2025). Proton Storage Chemistry in Aqueous Zinc-Inorganic Batteries with Moderate Electrolytes. Adv. Mater..

[cit17] Tleukenov Y. T., Kalimuldina G., Arinova A., Issatayev N., Bakenov Z., Nurpeissova A. (2022). Polyacrylonitrile-polyvinyl alcohol-based composite gel-polymer electrolyte for all-solid-state lithium-ion batteries. Polymers.

[cit18] Issatayev N., Adylkhanova A., Salah M., Bakenov Z., Kalimuldina G. (2024). Room temperature growth of NiS hierarchical nanoflowers on the flexible electrode surface as a cathode for lithium-ion batteries. Mater. Lett..

[cit19] Zhao L., Liu X., Li J., Diao X., Zhang J. (2023). One–Step Synthesis of Three–Dimensional Na3V2 (PO4) 3/Carbon Frameworks as Promising Sodium–Ion Battery Cathode. Nanomaterials.

[cit20] He Y., Li H. Recent research process of carbon engineering on Na3V2 (PO4) 3 for sodium-ion battery cathodes: A mini review. Electron. Mater..

[cit21] Li X., Wang S., Tang X., Zang R., Li P., Li P., Man Z., Li C., Liu S., Wu Y., Wang G. (2019). Porous Na3V2 (PO4) 3/C nanoplates for high-performance sodium storage. J. Colloid Interface Sci..

[cit22] Yang J., Zhang J., Lau V. W., Park M., Lee S., Kim J., Kang Y. M. (2020). Interface-controlled rhombohedral Li3V2 (PO4) 3 embedded in carbon nanofibers with ultrafast kinetics for Li-ion batteries. J. Phys. Chem. Lett..

[cit23] Qin R., Wei Y., Zhai T., Li H. (2018). LISICON structured Li 3 V 2 (PO 4) 3 with high rate and ultralong life for low-temperature lithium-ion batteries. J. Mater. Chem. A.

[cit24] Mukushev I., Tyan Y., Kalimuldina G., Mukanova A., Jakupova Z., Kim S. S., Bakenov Z., Nurpeissova A. (2025). High-performance Na3V2 (PO4) 3/C cathode for efficient low-temperature lithium-ion batteries. NPG Asia Mater..

[cit25] Zhao N., Li Y., Zhao X., Zhi X., Liang G. (2016). Effect of particle size and purity on the low temperature electrochemical performance of LiFePO4/C cathode material. J. Alloys Compd..

[cit26] Kate R. S., Kadam S. V., Kulkarni M. V., Deokate R. J., Kale B. B., Kalubarme R. S. (2023). Highly stable and nanoporous Na3V2 (PO4) 3@ C cathode material for sodium-ion batteries using thermal management. J. Energy Storage.

[cit27] Boutelle E., Chen A., Gopal R., Bai P. (2025). One-Pot Aqueous Synthesis of Hierarchical Na3V2 (PO4) 3 Particles for High-Performance Sodium Batteries. J. Electrochem. Soc..

[cit28] Issatayev N., Kalimuldina G., Nurpeissova A., Bakenov Z. (2021). Biomass-derived porous carbon from agar as an anode material for lithium-ion batteries. Nanomaterials.

[cit29] Tietz F., Soundaraj P. V., Dashjav E., Grüner D., Prünte S., Dellen C. (2024). Influence of Carbon Content on the Ionic and Electronic Conductivities of Dense Nvp/C Composites. J. Power Sources Adv..

[cit30] Issatayev N., Abdumutaliyeva D., Tashenov Y., Yeskozha D., Seipiyev A., Bakenov Z., Nurpeissova A. (2024). Three-dimensional carbon coated and high mass-loaded NiO@ Ni foam anode with high specific capacity for lithium ion batteries. RSC Adv..

[cit31] He J., Tao T., Yang F., Sun Z. (2022). Unravelling Li+ Intercalation Mechanism and Cathode Electrolyte Interphase of Na3V2 (PO4) 3 and Na3 (VOPO4) 2F Cathode as Robust Framework Towards High-Performance Lithium-Ion Batteries. ChemSusChem.

[cit32] Ihsan-Ul-Haq M., Cui J., Mubarak N., Xu M., Shen X., Luo Z., Huang B., Kim J. K. (2021). Revealing cathode–electrolyte interface on flower-shaped Na3V2 (PO4) 3/C cathode through cryogenic electron Microscopy. Adv. Energy Sustain. Res..

[cit33] Bi L., Li X., Liu X., Zheng Q., Lin D. (2019). Enhanced cycling stability and rate capability in a La-doped Na3V2 (PO4) 3/C cathode for high-performance sodium ion batteries. ACS Sustain. Chem. Eng..

[cit34] Wang M., Wang Q., Ding X., Wang Y., Xin Y., Singh P., Wu F., Gao H. (2022). The prospect and challenges of sodium-ion batteries for low-temperature conditions. Interdiscip. Mater..

[cit35] Jian Z., Zhao L., Pan H., Hu Y. S., Li H., Chen W., Chen L. (2012). Carbon coated Na3V2 (PO4) 3 as novel electrode material for sodium ion batteries. Electrochem. Commun..

[cit36] Yang J., Choi D., Kim K. S., Kim D. U., Kim J. (2021). Poly (vinylalcohol)(pva) assisted sol-gel fabrication of porous carbon network-Na3V2 (PO4) 3 (NVP) composites cathode for enhanced kinetics in sodium ion batteries. Polymers.

[cit37] Shen W., Wang C., Liu H., Yang W. (2013). Towards highly stable storage of sodium ions: a porous Na3V2 (PO4) 3/C cathode material for sodium-ion batteries. Chem.–Eur. J..

[cit38] Chen Y., Guo J., Liu Y., Zhang W., Chen C., Zhou H., Liao Y., Xing L., Li W. (2025). Exploring the genuine effect of fluoroethylene carbonate on cathode in virtue of sodium vanadium phosphate. J. Power Sources.

[cit39] Belgibayeva A., Kydyrbayeva U., Rakhatkyzy M., Kalimuldina G., Nurpeissova A., Bakenov Z. (2025). Low-temperature performance of Zn-modified graphite and hard carbon as anodes for lithium-ion batteries. Solid State Sci..

[cit40] Belgibayeva A., Rakhmetova A., Rakhatkyzy M., Kairova M., Mukushev I., Issatayev N., Kalimuldina G., Nurpeissova A., Sun Y. K., Bakenov Z. (2023). Lithium-ion batteries for low-temperature applications: Limiting factors and solutions. J. Power Sources.

